# Acoustic Fabrication of Collagen–Fibronectin Composite Gels Accelerates Microtissue Formation

**DOI:** 10.3390/app10082907

**Published:** 2020-04-23

**Authors:** Emma G. Norris, Diane Dalecki, Denise C. Hocking

**Affiliations:** 1 Department of Pharmacology and Physiology, University of Rochester, Rochester, NY 14642, USA;; 2 Department of Biomedical Engineering, University of Rochester, Rochester, NY 14627, USA;

**Keywords:** ultrasound, collagen, fibronectin, hydrogel, tissue engineering, acoustics, biofabrication

## Abstract

Ultrasound can influence biological systems through several distinct acoustic mechanisms that can be manipulated by varying reaction conditions and acoustic exposure parameters. We recently reported a new ultrasound-based fabrication technology that exploits the ability of ultrasound to generate localized mechanical forces and thermal effects to control collagen fiber microstructure non-invasively. Exposing solutions of type I collagen to ultrasound during the period of microfibril assembly produced changes in collagen fiber structure and alignment, and increased the biological activity of the resultant collagen hydrogels. In the extracellular matrix, interactions between fibronectin and collagen fibrils influence the biological activity of both proteins. Thus, in the present study, we examined how addition of fibronectin to collagen solutions prior to ultrasound exposure affects protein organization and the biological activity of the composite hydrogels. Results indicate that ultrasound can alter the distribution of fibronectin within 3D hydrogels via thermal and non-thermal mechanisms to produce composite hydrogels that support accelerated microtissue formation. The use of acoustic energy to drive changes in protein conformation to functionalize biomaterials has much potential as a unique, non-invasive technology for tissue engineering and regenerative medicine.

## 1. Introduction

The development of biocompatible scaffolds that can coordinate complex, multicellular behaviors within artificial environments is essential for the development of tissue-engineered materials for a variety of research applications and clinical needs, including cutaneous healing in patients affected by chronic and hard-to-heal wounds [1,2]. Timely closure of cutaneous wounds requires the coordinated response of multiple cell types, each executing various behaviors [3]. A critical event during wound healing is the remodeling of the extracellular matrix (ECM) which occurs through processes of matrix proteolysis, deposition, and contraction [3,4]. Dysregulation of these tightly controlled processes occurs in many chronic illnesses including diabetes and vascular disease, and results in the failure of wounds to heal [4]. Despite significant innovations in developing bioactive dressings to support or enhance wound healing, the complex, multifactorial nature of chronic wounds has remained a persistent challenge [5]. As a result, as many as 40–50% of patients who are treated for chronic wounds annually fail to achieve complete wound closure [6,7].

Collagen and fibronectin are two major components of the cutaneous ECM, and both play essential roles in dermal wound healing. Type I collagen is the most abundant protein in the human body [8], and is the predominant component of scar tissue produced as a result of successful wound closure [3]. Collagen-based dressings are widely used in clinical environments for the treatment of chronic and non-healing wounds [9]. The versatility of collagen within tissue engineering environments derives, in part, from its self-assembly capacity [9]. As well, techniques for purifying large quantities of collagen and fabricating three-dimensional (3D) hydrogels are well established [10,11]. However, many collagen-based wound dressings rely on in vitro culture with allogenic cells to generate materials with appropriate mechanical and biological properties for wound healing [9]. This labor-intensive manufacturing process has been a significant barrier to commercialization, and a substantial economic burden for healthcare systems [12]. Therefore, methods to enhance the regenerative capacity of collagenous biomaterials are an area of active research and critical importance [11].

Fibronectin is a large, dimeric glycoprotein that is assembled into insoluble ECM fibers via a cell-mediated process that is dependent on interactions with cell surface receptors in combination with cell-derived tension [13]. Importantly, binding interactions between collagen and fibronectin regulate the structure and function of both proteins. During cell-mediated ECM assembly, collagen and fibronectin are co-deposited into fibrils, and loss of either protein can impair the deposition of the other [14–16]. Furthermore, inhibition or deletion of fibronectin’s collagen-binding domain attenuates the ability of ECM fibronectin to stimulate cell behaviors critical to wound healing, including migration and proliferation [17,18]. Importantly, cell-mediated fibronectin fibril formation is also required for collagen fiber remodeling [19,20]. Therefore, technologies that replace cell-derived tension on fibronectin with an external source of mechanical force may provide a cost-effective alternative to cell-embedded collagen scaffolds in regenerative medicine applications.

Ultrasound is emerging as a versatile technique for manipulating scaffold structure within tissue-engineered environments, as it can be applied non-invasively and site-specifically within a variety of experimental and clinical contexts. Ultrasound is capable of influencing biological systems through several distinct mechanisms, which include both ultrasound-induced heating and the generation of mechanical force [21]. Thus, ultrasound-based technologies are particularly suited to interventions targeting collagen and fibronectin, both of which are sensitive to thermal and mechanical stimuli [22–26].

Previous work established that ultrasound exposure during collagen polymerization produces changes in collagen fiber organization within 3D collagen hydrogels via both thermal [27] and non-thermal mechanisms [28]. In turn, ultrasound-induced changes in collagen microstructure gave rise to enhanced cell functions, including increased cell migration, reduced adhesion strength, and increased collagen fiber remodeling [28]. In the present study, we investigated effects of ultrasound on the structure and function of collagen and fibronectin co-polymerized within 3D hydrogels. Additionally, we assessed the ability of acoustically modified composite hydrogels to influence the progression of cellular self-assembly, as an in vitro marker of coordinated cell behaviors involved in tissue formation. The results of this study indicate that ultrasound can influence the distribution of fibronectin within 3D hydrogels via both thermal and non-thermal mechanisms to produce acoustically modified composite hydrogels that support accelerated microtissue formation.

## 2. Materials and Methods

### 2.1. Generation and Characterization of Acoustic Fields

**Figure A1.**
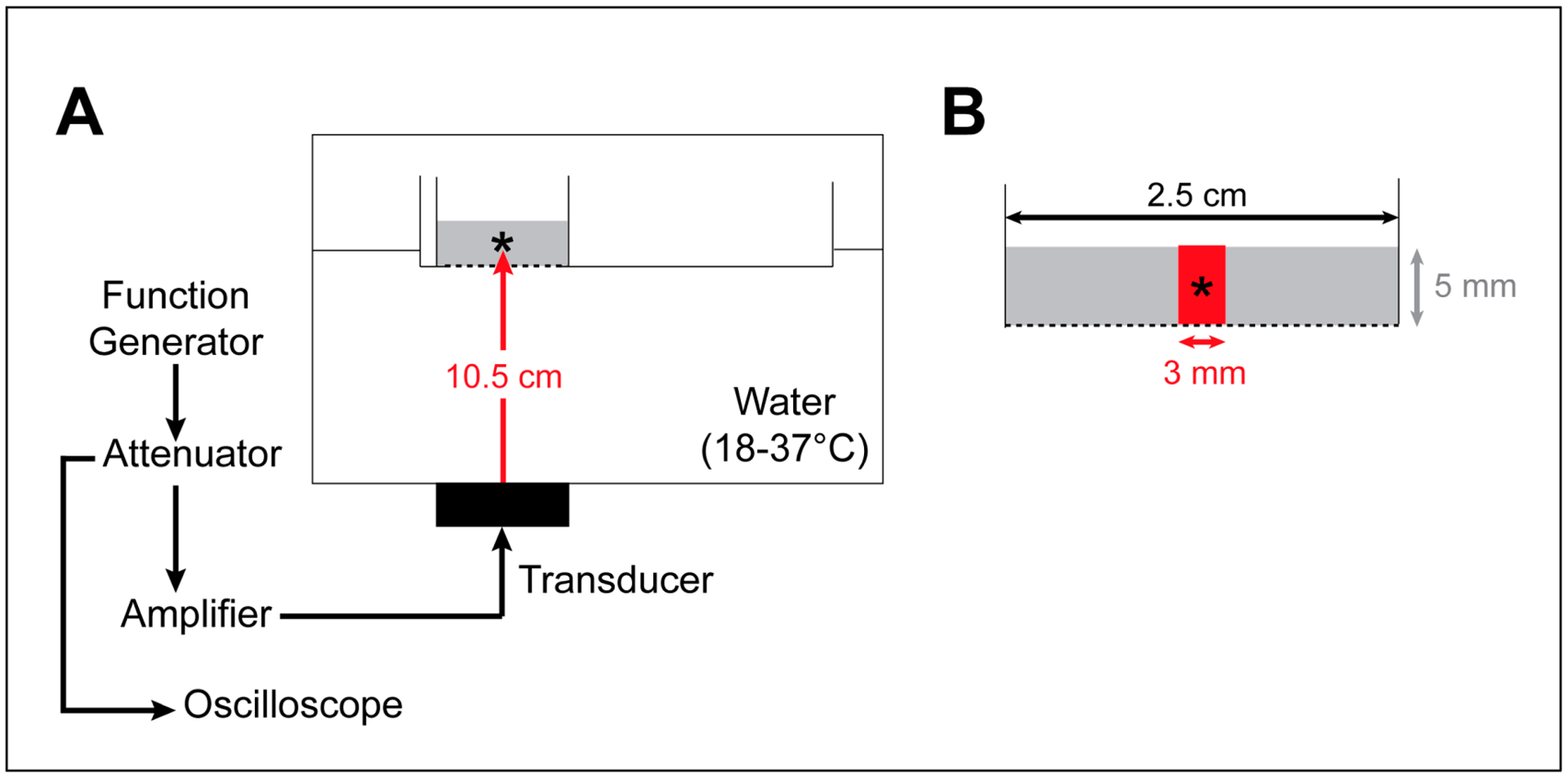
Schematic of acoustic exposure system for fabrication of collagen–fibronectin composite hydrogels. (**A**) Ultrasound fields (8.8 MHz, 3.8 or 8.2 W/cm^2^ I_SPPA_) were generated using an unfocused piezoceramic transducer (1-cm diameter) mounted at the bottom of a temperature-controlled water tank. Ultrasound fields were calibrated in the free field (asterisk) prior to acoustic exposures of samples at a location 10.5 cm away from the transducer face (red arrow). Exposures were performed by lowering the water level and placing a modified tissue culture plate with a flexible elastomer membrane bottom (dashed line) at the surface of the water tank such that the center (asterisk) of the hydrogel sample (gray box) was positioned at the calibration location. (**B**) Cross-sectional scale diagram illustrating dimensions of the acoustic exposure well. Red box indicates the half-maximal acoustic beam width (3 mm). Asterisk indicates location of acoustic field calibration, positioned at the center of the sample. The sample volume was calculated to form a fluid height of 5 mm and the total well diameter was 2.5 cm.

Ultrasound fields were generated as described previously [28] and the acoustic exposure set-up is illustrated in Figure A1 (Appendix A). Briefly, a 1-cm diameter unfocused piezoceramic transducer was mounted at the bottom of a plastic exposure tank filled with degassed, deionized water. The transducer was driven by a continuous sinusoidal signal at its fundamental frequency (8.8 MHz) using a function generator (AFG3022B; Tektronix, Beaverton, OR, USA,), attenuator (837; KayPENTAX, Montvale, NJ, USA), and RF power amplifier (2100L; ENI, Rochester, NY, USA). Acoustic fields were characterized using both needle (HNC-0400; Onda, Sunnyvale, CA, USA) and capsule (HGL-0085; Onda) hydrophones. A location in the far field was selected (10.5 cm from transducer) such that the transaxial beam width was 3 mm. Acoustic fields were calibrated in the free field before and after each experiment for both amplitude (peak positive and peak negative pressure, MPa) and spatial peak pulse average intensity (I_SPPA_, W/cm^2^). Values from each calibration were averaged across all experiments and are reported as mean ± SEM in Table 1.

### 2.2. Fabrication of Composite Collagen–Fibronectin Hydrogels

Collagen hydrogels were polymerized in the presence of ultrasound, as described previously [28] with the following modifications. Neutralized collagen solutions were prepared by combining equal volumes of type I rat tail collagen (Corning, Lowell, MA, USA) with 2X Dulbecco’s modified Eagle medium (DMEM; Invitrogen, Carlsbad, CA, USA). Collagen concentration was adjusted to 2 mg/mL by addition of fibroblast culture media (1:1 mixture of AimV (Invitrogen) and SF Medium (Corning) containing 25 mM HEPES). The pH of the solution was adjusted to 7.4 by addition of 0.1 N NaOH. Collagen solutions were maintained on ice until gel fabrication. Fibronectin was purified from outdated human plasma (American Red Cross, Rochester, NY, USA) using gelatin-Sepharose (GE Healthcare, New York, NY, USA) affinity chromatography, as described [18]. Fibronectin (25 μg/mL final concentration) was added to soluble, neutralized collagen immediately prior to ultrasound exposure.

Collagen–fibronectin hydrogels were fabricated within one well of a modified elastomer-bottomed tissue culture plate. Plates were placed within the acoustic exposure tank using a three-axis positioner (Velmex, Leatherhead, UK, Series B4000 Unislide) such that the center of the well was in contact with the water bath and the acoustic beam was centered within the well. The temperature of the water in the exposure tank was set to 18, 25, or 37 °C. Hydrogel polymerization was initiated in response to the temperature rise in the sample upon transferring collagen–fibronectin solutions to the exposure well [29]. Samples were exposed to continuous wave ultrasound for 15 min, which was sufficient time for the sample to transition from a fluid solution to a solid hydrogel. Sham-exposed hydrogels were fabricated using the same exposure system and identical protocols, but the ultrasound was not activated. Collagen–fibronectin hydrogels were placed in a humidified incubator (37 °C, 8% CO_2_) overnight to ensure complete collagen polymerization before subsequent experiments.

### 2.3. Multiphoton Microscopy

Alexa546-labeled fibronectin (AF546-FN) was prepared by incubating plasma fibronectin with Alexa Fluor 546 tetrafluorophenyl (TFP) ester (Thermo Fisher Scientific, Waltham, MA, USA) according to manufacturer’s instructions; unreacted dye was removed by size exclusion chromatography [30]. Unlabeled fibronectin (22.5 μg/mL) and AF546-FN (2.5 μg/mL) were added to aliquots of collagen (2 mg/mL) immediately prior to co-polymerization under ultrasound- or sham-exposure conditions. Polymerized gels were washed with phosphate-buffered saline (PBS) to remove unbound fibronectin and fixed in 2% paraformaldehyde in PBS for 1 h prior to multiphoton imaging.

Multiphoton images were collected using a FVMPE-RS microscope equipped with a 25X (NA 1.05) objective (Olympus Scientific, Waltham, MA, USA). Samples were illuminated with 800-nm light generated by a Mai Tai HP Deep See Ti:Sa laser (Spectra-Physics, Santa Clara, CA, USA). Collagen fibers were visualized using second harmonic generation (SHG). Emitted light was detected with a photomultiplier tube through bandpass filters of either 370–410 nm (collagen, SHG) or 575–630 nm (AF546-FN). Images were acquired through a depth of 100 μm in 5-μm z-steps beginning at the surface of the gel. Representative images were acquired from at least 3 positions within a 0.5-cm diameter area of interest corresponding to the center of each gel. Maximum intensity axial projections were reconstructed using FIJI software (National Institutes of Health).

### 2.4. Temperature Measurements

The maximum temperature achieved within the center of the hydrogel during fabrication was measured using type-T wire thermocouples as described previously [28]. Briefly, thermocouples were placed within exposure plates such that the junction was positioned within the acoustic beam and 2.5 mm above the elastomer membrane bottom. Collagen solutions (2 mg/mL in DMEM) were added and exposed to ultrasound or sham conditions for 15 min in the exposure tank with the water temperature set to 18, 25, or 37 °C. Gel temperature was recorded every 15 s for the duration of the exposure. Peak temperatures reported in Table 1 are the mean temperature averaged across the final minute of exposure, at which point equilibrium temperature had been reached.

### 2.5. Microtissue Formation Assay

Fibronectin-null mouse embryonic fibroblasts (FN-null MEFs) were cultured on tissue culture flasks pre-coated with type I rat tail collagen, using a 1:1 mixture of AimV and SF Medium [28]. These media do not contain fibronectin and do require serum-supplementation. FN-null MEFs do not produce fibronectin endogenously, but can assemble fibronectin and collagen fibrils when provided with an exogenous source of fibronectin, allowing for precise control over the matrix assembly process [31]. Cells were seeded at a density of 4 × 10^4^ cells/cm^2^ onto the surface of collagen–fibronectin gels polymerized under ultrasound- or sham-exposure conditions. Cell-seeded collagen gels were incubated at 37 °C, 8% CO_2_ for up to 6 days. The formation of microtissues was monitored using phase-contrast microscopy (BX-60 microscope, Olympus, Tokyo, Japan) at 24 h, 3 d, and 6 d post-seeding. Images were obtained using a digital camera (Teledyne Imaging, Tucson, AZ, USA). At each time point, images were collected beginning at the region of the gel corresponding to the center of the ultrasound exposure and moving off-center in non-overlapping steps (1.6 mm).

Microtissue formation was analyzed within each region of interest (ROI, 2.2 × 1.6 mm) using FIJI software. The number of microtissues was quantified as the number of independent cell clusters consisting of 3 or more cells. If cell clusters were connected to one or more nearby cell clusters via a cellular network, only the largest cluster was counted. As a second measure of cellular self-assembly, the surface area occupied by cells within each ROI was measured by manually tracing the edges of both adherent cell monolayers and 3D microtissues. Individual cells and groups containing fewer than 3 cells were excluded from the measurement.

### 2.6. Adhesion Assay

FN-null MEFs were seeded at a density of 2 × 10^4^ cells/cm^2^ onto collagen–fibronectin gels polymerized under ultrasound- or sham-exposure conditions [32]. Cell-seeded gels were incubated for 10 min at 37 °C, 8% CO_2_, and then washed with PBS to remove non-adherent cells. Phase-contrast microscopy was used to capture images of a ROI (2.2 × 1.6 mm) corresponding to the center of the acoustic exposure area. The number of adherent cells within each image was quantified using FIJI software.

### 2.7. Statistical Analyses

All statistical analyses were performed using Prism software (Version 8, GraphPad, San Diego, CA, USA) and are presented as mean ± SEM for *n* ≥ 3 samples per condition and fabricated on at least 3 independent days. Mean values for each condition were compared using one-way analysis of variance (ANOVA) followed by Bonferroni’s post-hoc test. Repeated measures ANOVA was used to match comparisons involving multiple locations across the surface of the same gel. Effects of polymerization temperature were analyzed by comparing temperature-matched pairs using a two-tailed t-test. *p* values <0.05 were considered statistically significant.

## 3. Results

### 3.1. Ultrasound Exposure Produces Changes in Collagen–Fibronectin Fiber Structure that Support Accelerated Microtissue Formation

**Figure 1.**
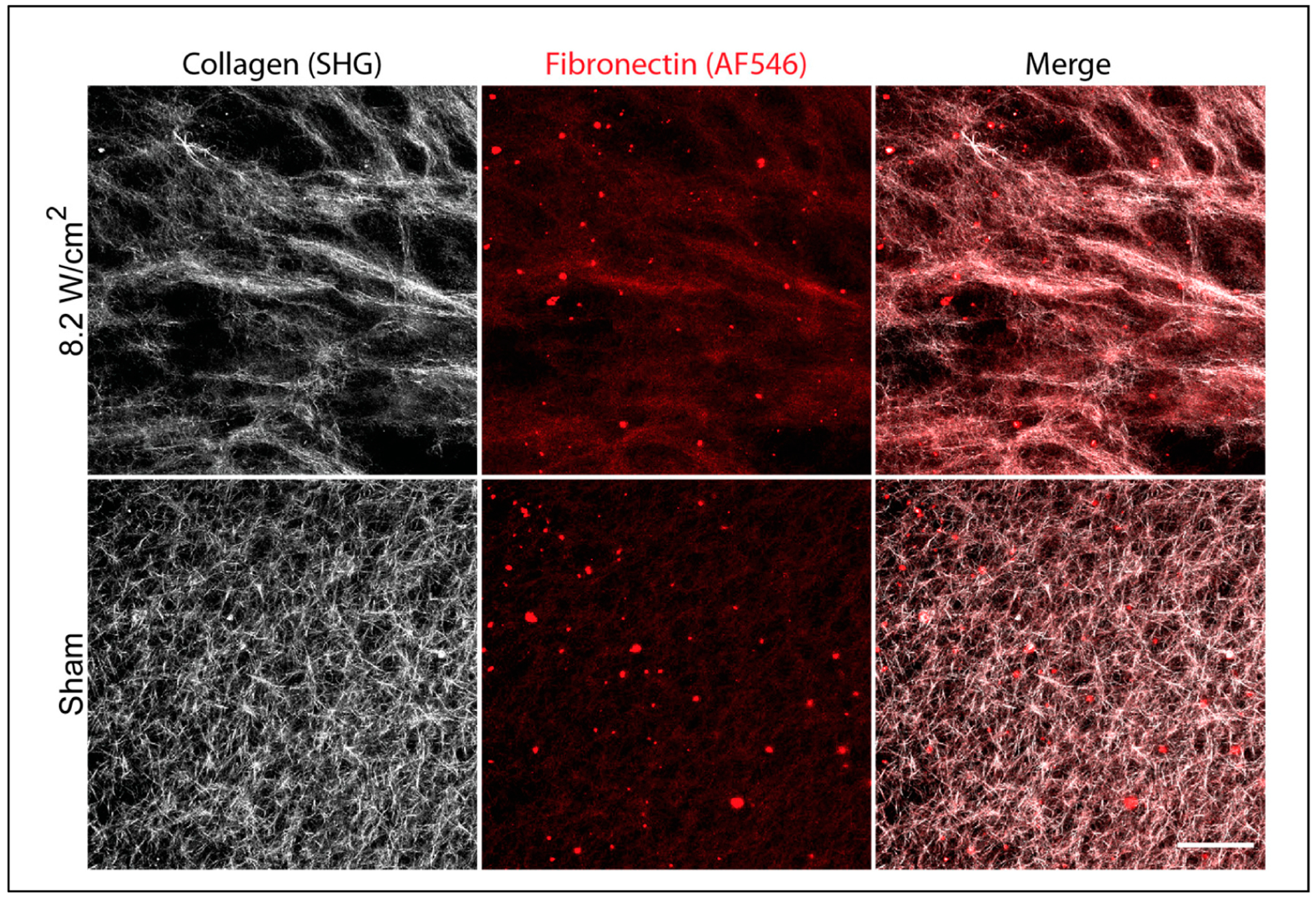
Effects of ultrasound on fibril structure in collagen–fibronectin composite gels. Neutralized solutions of type I collagen (2 mg/mL) and fibronectin (25 μg/mL, 10% AF546-labeled) were co-exposed to either 8.8-MHz ultrasound at an intensity of 8.2 W/cm^2^ or sham conditions (0 W/cm^2^) in a 25 °C water bath. Unbound fibronectin was removed by media exchange prior to fixation and multiphoton imaging of collagen (white) and fibronectin (red) fiber structures. Maximum intensity z-projection images through a depth of 15 μm (5-μm z-steps) at the gel surface were assembled using FIJI software. Shown are representative images from 1 of 3 samples per condition fabricated on independent days. Scale bar = 100 μm.

In previous work, we demonstrated that ultrasound exposure during collagen hydrogel formation can produce functional changes in the organization of collagen fibers [28]. However, in healing dermal wounds, fibronectin often co-localizes with collagen fibers [33] and collagen fibril formation and remodeling by cells requires fibronectin-collagen interactions [15,20]. Thus, composite hydrogels, containing both collagen and fibronectin, were fabricated in the presence and absence of ultrasound, and then imaged by multiphoton microscopy. As observed previously [28], ultrasound-exposed collagen gels contained regions of dense, radially aligned collagen fibers (Figure 1, 8.2 W/cm^2^, SHG). Co-staining for fibronectin revealed diffuse co-localization with collagen fibrils (Figure 1, Merge); individual fibronectin fibrils were not detected (Figure 1, 8.2 W/cm^2^, AF546-FN). Composite hydrogels polymerized under sham conditions contained short, randomly oriented collagen fibers (Figure 1, Sham, SHG) and exhibited some, albeit limited fibronectin staining (Figure 1, Sham, AF546-FN). No differences were observed in the overall intensity of fibronectin staining on ultrasound-exposed compared to sham-polymerized hydrogels (Figure 1, AF546). However, changes in fibronectin organization were observed that were consistent with the corresponding collagen fiber structures.

Fibronectin and collagen co-regulate a variety of cell behaviors involved in tissue formation, including migration, proliferation, and matrix contraction [17]. Previous work characterizing the behavior of mesenchymal cells in collagen- and fibronectin-containing cell culture systems demonstrated that on native collagen gels and in the presence of fibronectin, FN-null MEFs [18] and dermal fibroblasts [34] can self-assemble to form 3D multicellular spheroids or “microtissues”, by a process that requires fibronectin fibril assembly. Microtissue formation is further influenced by collagen–fibronectin interactions [18], substrate adhesive properties, and cellular contractility [35]. As such, cohesion of FN-null MEFs into 3D multicellular structures is a useful tool for assessing the ability of fibronectin/collagen-based hydrogels to support a coordinated cellular response.

**Figure 2.**
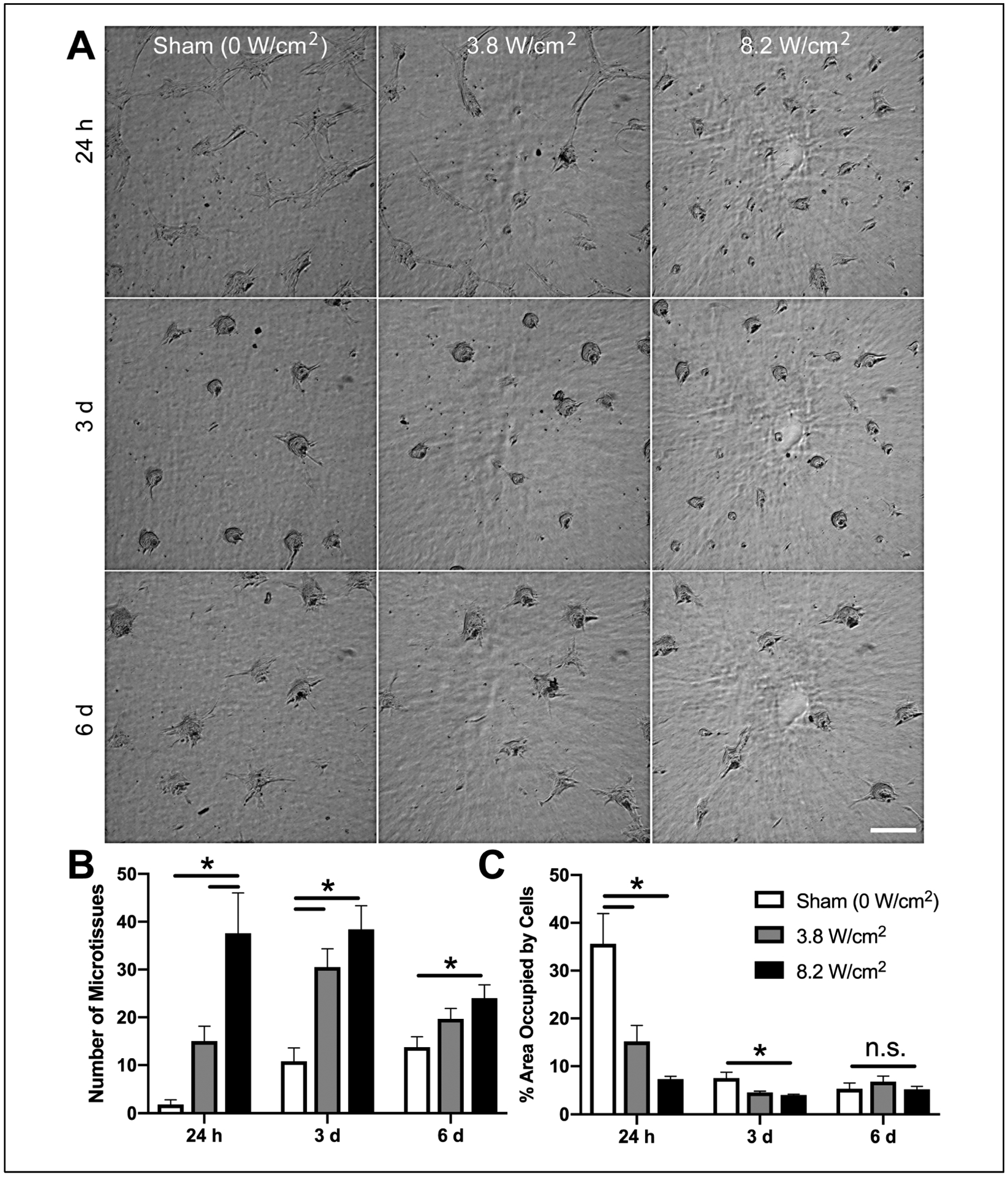
Acoustically modified collagen–fibronectin composite gels accelerate microtissue formation. Neutralized solutions of type I collagen (2 mg/mL) and fibronectin (25 μg/mL) were co-exposed to 8.8-MHz ultrasound or sham conditions during gel polymerization in a 25 °C water tank. Acoustic intensities were calibrated in the free field for 0 (sham), 3.8, or 8.2 W/cm^2^. FN-null MEFs (4×10^4^ cells/cm^2^) were seeded on the surface of polymerized gels and cultured for up to 6 d. (**A**) Phase-contrast images were collected from the region of the gel corresponding to the center of the acoustic exposure at 24 h, 3 d, and 6 d post-seeding. Scale bar = 200 μm. (**B**) The number of microtissues at each time point was quantified for each polymerization condition by counting the number of isolated cell clusters within a 3.7 mm^2^ ROI corresponding to the center of the acoustic exposure. (**C**) The area occupied by cells in each ROI was quantified by manually tracing the outlines of cell groups and is presented as the percentage of the ROI surface area occupied by cells. Data are presented as mean ± SEM for *n* ≥ 4 gels fabricated on independent days. Sham, white; 3.8 W/cm^2^, gray; 8.2 W/cm^2^, black. Significantly different means, * *p* < 0.05 by one-way ANOVA with Bonferroni’s post-hoc test.

Cells cultured on sham-exposed, collagen–fibronectin hydrogels formed tightly adherent, well-spread monolayers across the gel surface within the first 24 h of culture (Figure 2A, Sham, 24 h). As expected [18], after 3 days of culture, cells had coalesced into 3D multicellular spheroids (Figure 2A, Sham, 3 d), termed “microtissues”. At 24 h, the morphology of cells cultured on collagen–fibronectin gels fabricated in the presence of 8.8-MHz ultrasound at the lower intensity of 3.8 W/cm^2^ was similar to that of cells on sham-exposed gels (Figure 2A, 3.8 W/cm^2^, 24 h). In contrast, cells cultured on the surface of collagen–fibronectin gels fabricated using 8.8-MHz ultrasound at the higher intensity of 8.2 W/cm^2^ formed numerous small microtissues within the first 24 h of culture (Figure 2A,B, 8.2 W/cm^2^). By day 3, significantly more microtissues had formed on ultrasound-fabricated versus sham-exposed gels (Figure 2A,B, 3 d). Between days 3 and 6, the number of microtissues on ultrasound-exposed gels decreased as some of the microtissues coalesced; however the total number of microtissues on gels fabricated using ultrasound at 8.2 W/cm^2^ remained significantly greater than sham-exposed gels (Figure 2B, 6 d).

**Figure 3.**
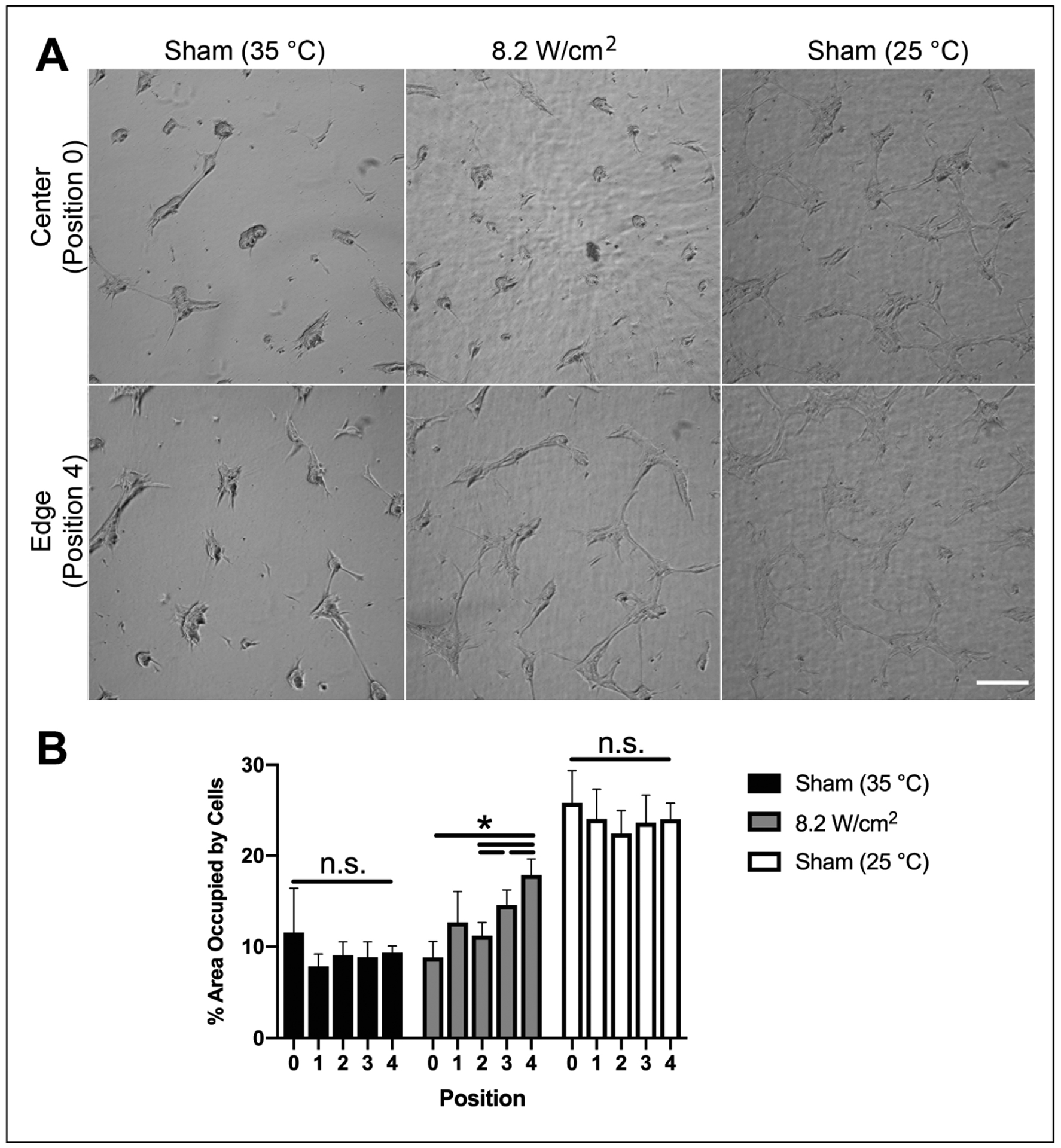
Accelerated microtissue formation on acoustically modified gels is localized to the acoustic beam width and dependent on polymerization temperature. Neutralized solutions of type I collagen (2 mg/mL) and fibronectin (25 μg/mL) were co-exposed to 8.8-MHz ultrasound at an intensity of 8.2 W/cm^2^ in a 25 °C water tank. Sham-exposed samples were polymerized with the water temperature set to either 25 or 37 °C to achieve polymerization temperatures corresponding to the temperature achieved at the center (32 °C) or edge (25 °C) of the ultrasound-exposed gel. FN-null MEFs (4 × 10^4^ cells/cm^2^) were seeded on the surfaces of polymerized hydrogels and cultured for 24 h. (**A**) Phase-contrast images were collected beginning at a position corresponding to the acoustic exposure location (position 0), and moving off-center radially in 1.6 mm steps to the edge of the gel (positions 1–4). Scale bar = 200 μm. (**B**) The area occupied by cells within each ROI was quantified by tracing the outlines of cell clusters and measuring the percentage of the surface area occupied by cells. Data are presented as mean ± SEM for *n* = 4 gels fabricated on independent days. Significantly different means, * *p* < 0.05 by repeated measures ANOVA with Bonferroni’s post-hoc test.

As a second measure of cellular self-assembly, the area of the gel surface occupied by cells was quantified. Cells were maximally spread on sham-exposed gels at 24 h post-seeding, occupying ~35% of the gel surface (Figure 2C, Sham, 24 h). As microtissues formed on sham-exposed gels over the course of 6 days, cell-occupied area decreased (Figure 2C, Sham, 3 and 6 d). Similar to results shown in Figure 2B, there was a significant reduction in cell-occupied area at 24 h on ultrasound-fabricated versus sham-exposed gels (Figure 2C, 24 h). On day 3, there remained a small but significant difference still present between sham- versus ultrasound-fabricated gels (Figure 2B, 3 h; sham vs. 8.8 W/cm^2^). The fraction of the gel surface area occupied by cells serves as a simple and effective parameter for quantifying changes in cellular self-assembly. Thus, in subsequent studies, this parameter was used to quantify the effect of various fabrication conditions on the ability of collagen–fibronectin hydrogels to influence the self-assembly processes.

### 3.2. Effects of Ultrasound on Microtissue Assembly are Temperature-Dependent and Spatially Localized

Previous studies demonstrated that ultrasound can influence collagen structure and function through both thermal [27] and non-thermal mechanisms [28]. Thus, studies were conducted to determine whether the ability of ultrasound-fabricated collagen/fibronectin hydrogels to accelerate microtissue formation were mediated via thermal or non-thermal mechanisms. To do so, we first measured the temperature rise within collagen gels that occurred in response to the acoustic exposure conditions used in this study. Collagen gels polymerized in the presence of 8.8-MHz ultrasound at an intensity of 8.2 W/cm^2^ heated to a final temperature of 32.3 °C, representing a 7.7 °C increase above corresponding sham-exposures (Table 1). Sham-exposed collagen gels polymerized in a 37 °C water tank heated to a temperature of 34.5 °C by the end of the 15 min exposure period (Table 1). Therefore, in subsequent experiments, sham-exposed gels polymerized in a 37 °C water were included as temperature-matched control conditions to recapitulate the maximal temperature rise induced by ultrasound at an intensity of 8.2 W/cm^2^.

Ultrasound-induced heating within collagen samples is highest at the center of the beam and lower at locations outside of the half-maximal beam width [27]. Thus, we next asked whether microtissue formation on ultrasound-fabricated gels was spatially localized to the acoustic beam area. Cells that had adhered to the center of collagen–fibronectin gels fabricated in the presence of 8.8-MHz ultrasound (8.2 W/cm^2^) formed microtissues within 24 h of culture (Figure 3A, 8.2 W/cm^2^, center). In contrast, cells that had adhered to regions of the gel outside of the area affected by the acoustic beam formed interconnected adherent networks rather than 3D microtissues (Figure 3A, 8.2 W/cm^2^, edge). To quantify differences in microtissue formation as a function of acoustic beam width, cell-occupied area was determined for sham- and ultrasound-exposed gels within non-overlapping ROIs beginning at the center of the gel and moving off-center in 1.6-mm steps. On ultrasound-exposed gels, a gradient of microtissue formation occurred along the radius of the gel, with less than 10% cell-occupied area at the acoustic exposure location increasing to ~18% at the gel edge (Figure 3, 8.2 W/cm^2^, gray bars). Sham-exposed gels, polymerized at a temperature similar to that achieved at the center of the ultrasound beam, supported microtissue formation uniformly across the entire gel surface (Figure 3A, 35 °C Sham) and exhibited a similar cell-occupied area as the center ROI on ultrasound-exposed gels (Figure 3B). In contrast, cells seeded on sham-exposed gels, polymerized at a temperature comparable to that observed outside of the acoustic beam, did not form microtissues and (Figure 3A, 25 °C Sham) and occupied greater than 20% of the surface area at all locations (Figure 3B). Together, these data suggest that changes in collagen–fibronectin gel structure that support accelerated microtissue formation are mediated through a thermal acoustic mechanism, and furthermore, are spatially localized to regions within and immediately adjacent to the half-maximal acoustic beam width.

### 3.3. Acoustic Modification Reduces Initial Adhesion to Collagen–Fibronectin Hydrogels

**Figure 4.**
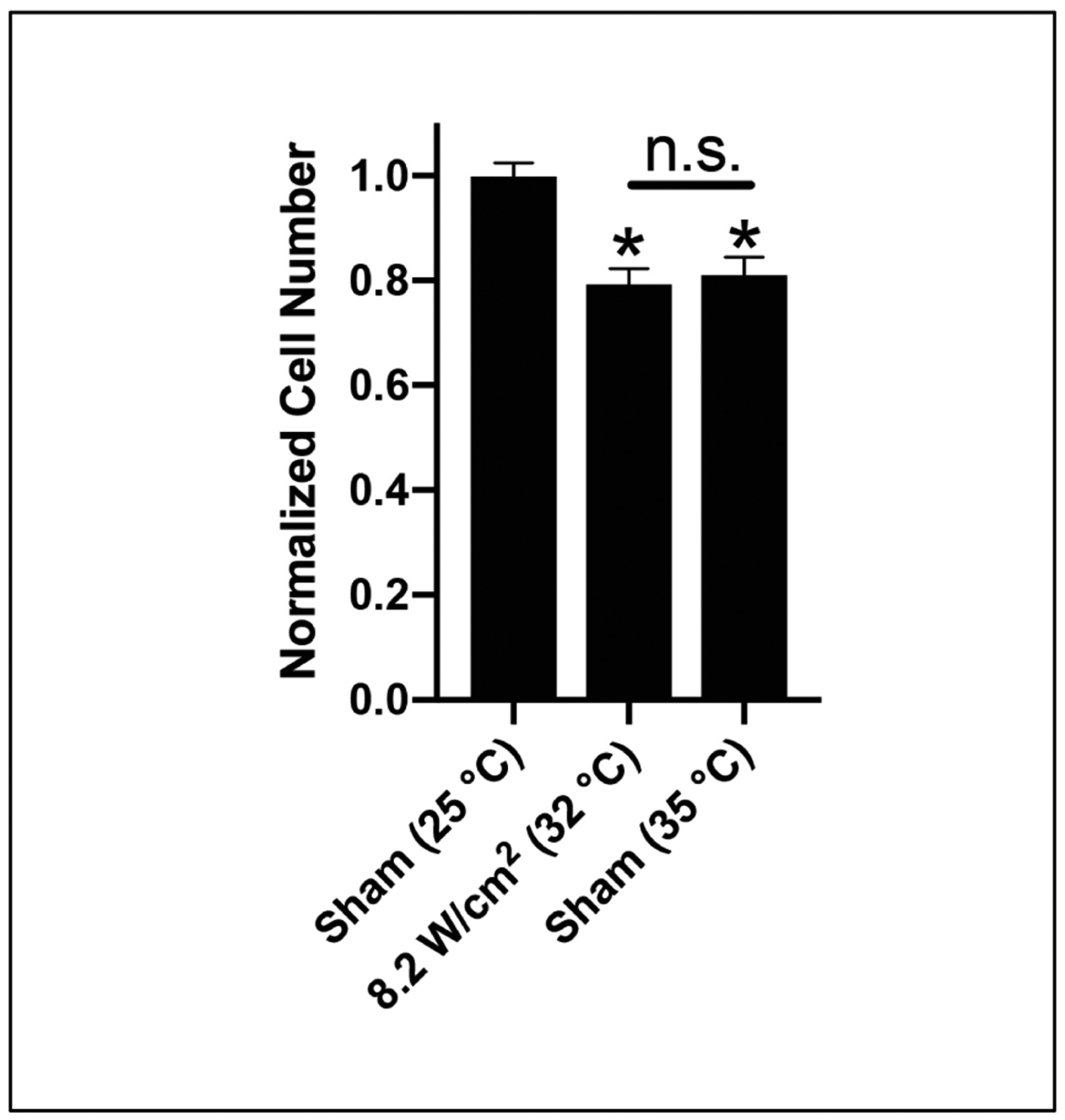
Ultrasound exposure decreases initial cell attachment strength. Neutralized solutions of collagen (2 mg/mL) and fibronectin (25 μg/mL) were co-polymerized during exposure to 8.8-MHz ultrasound at an intensity of 8.2 W/cm^2^ in a 25 °C water bath, or polymerized under sham conditions in a heated water bath to achieve polymerization temperatures corresponding to the temperature achieved at the center or edge of the ultrasound-exposed gel. The peak temperature achieved within the gel sample under each set of polymerization conditions is indicated in parentheses. FN-null MEFs (2 × 10^4^ cells/cm^2^) were seeded onto polymerized gels and allowed to adhere for 10 min before the number of adherent cells was determined. Data are presented as mean ± SEM for *n* ≥ 7 gels fabricated on 4 independent experimental days. Significantly different means, * *p* < 0.05 vs. 25 °C sham by one-way ANOVA with Bonferroni’s post-hoc test.

Cellular self-assembly in response to fibronectin is influenced, in part, by the adhesive properties of the substrate, wherein reduced adhesion strength correlates with enhanced microtissue assembly [35,36]. To determine whether ultrasound exposure affects adhesive properties of acoustically modified collagen–fibronectin hydrogels, cell adhesion assays were conducted. As shown in Figure 4, the number of adherent cells was significantly reduced on collagen–fibronectin gels fabricated using 8.8-MHz ultrasound at an intensity of 8.2 W/cm^2^ compared to sham-exposed gels polymerized at 25 °C (Figure 4, 25 °C sham vs. 8.2 W/cm^2^). A similar reduction in cell adhesion was observed on gels polymerized under sham conditions that mimicked the temperature rise induced by ultrasound exposure (Figure 4, 25 vs. 35 °C sham). These results are consistent with previous demonstrations that decreases in the initial cell-substrate binding strength are permissive for spheroidal microtissue formation [35], and provides further evidence that ultrasound exposure can accelerate microtissue formation via thermal effects on the collagen–fibronectin substrate.

### 3.4. Mechanical Effects of Ultrasound on Fibronectin Structure are Observed at Reduced Polymerization Temperature

**Figure 5.**
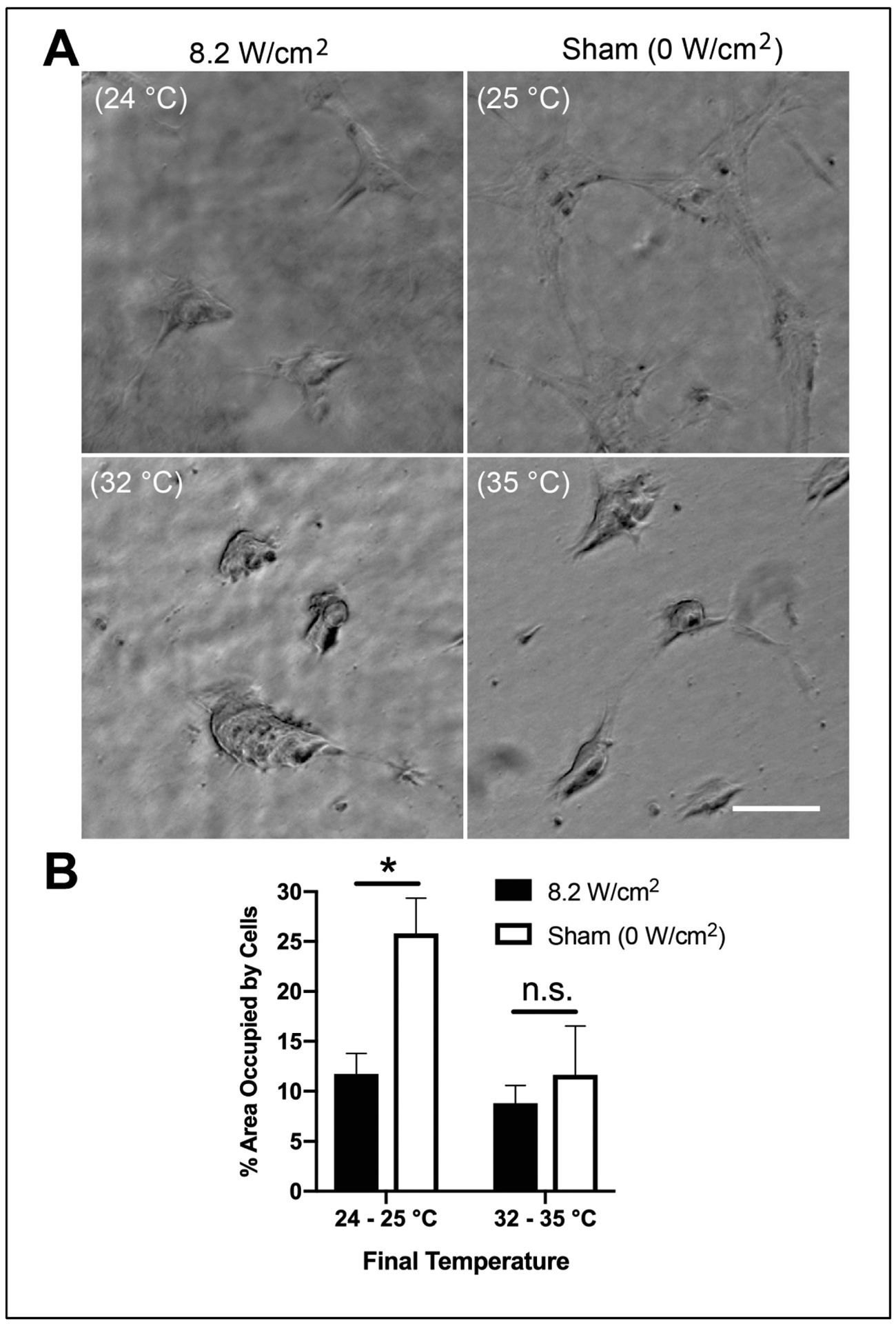
Mechanical and thermal effects of ultrasound can be uncoupled by reducing polymerization temperature. Neutralized solutions of type I collagen (2 mg/mL) and fibronectin (25 μg/mL) were exposed either to 8.8-MHz ultrasound at an intensity of 8.2 W/cm^2^, or sham-exposure conditions. The peak temperature achieved within the sample was adjusted by manipulating the water tank temperature and is reported for each condition in parentheses. FN-null MEFs (4 × 10^4^ cells/cm^2^) were seeded on the surfaces of fully polymerized hydrogels. (**A**) Phase-contrast images were collected 24 h post-seeding at a location corresponding to the center of the acoustic exposure. Scale bar = 100 μm. (**B**) The area occupied by cells within a ROI corresponding to the center of the gel was quantified by tracing the outlines of cell clusters and measuring the percentage of the cell-occupied surface area for ultrasound-exposed (black) and sham (white) gels for each polymerization condition. Data are presented as mean ± SEM for *n* ≥ 3 gels fabricated on independent days. Significantly different means, *p* < 0.05 by two-tailed t-test. n.s., not significant.

The conformations of both collagen and fibronectin are sensitive to temperature, as is the affinity of the interaction between the two proteins [22,23,37]. As temperatures are reduced from 33 to 30 °C, intact fibronectin undergoes a conformational change that increases its affinity for collagen by ~10-fold [22]. Therefore, we next performed experiments to investigate the bioactivity of acoustically modified collagen–fibronectin hydrogels fabricated under temperature conditions that were not allowed to exceed 30 °C. To do so, collagen–fibronectin solutions were exposed to 8.8-MHz ultrasound (8.2 W/cm^2^) in a water tank cooled to 18 °C. Under these conditions, the peak temperature achieved within the gel sample was 24 °C, similar to the temperature achieved within a 25 °C water bath under sham-exposure conditions (Table 1). Cells cultured for 24 h on the surface of these reduced-temperature, ultrasound-exposed collagen–fibronectin gels assembled into elongated, tightly adherent microtissues (Figure 5A, 8.2 W/cm^2^, 24 °C). In contrast, cells cultured for 24 h on temperature-matched sham-exposed gels formed 2D interconnected networks (Figure 5A, 25 °C Sham). Quantification of cell-occupied area demonstrated a significant reduction in cell-occupied area on reduced-temperature, ultrasound-exposed gels compared to the corresponding, temperature-matched sham conditions (Figure 5B, 24–25 °C). In contrast, no difference in cell-occupied area was observed on the temperature-matched ultrasound and sham-exposed gels polymerized under conditions that exceeded 30 °C, wherein both gels supported microtissue formation (Figure 5B, 32–35 °C). Thus, at fabrication temperatures below ~30 °C, ultrasound-mediated effects on the functional properties of collagen–fibronectin hydrogels are mediated by non-thermal mechanisms.

**Figure 6.**
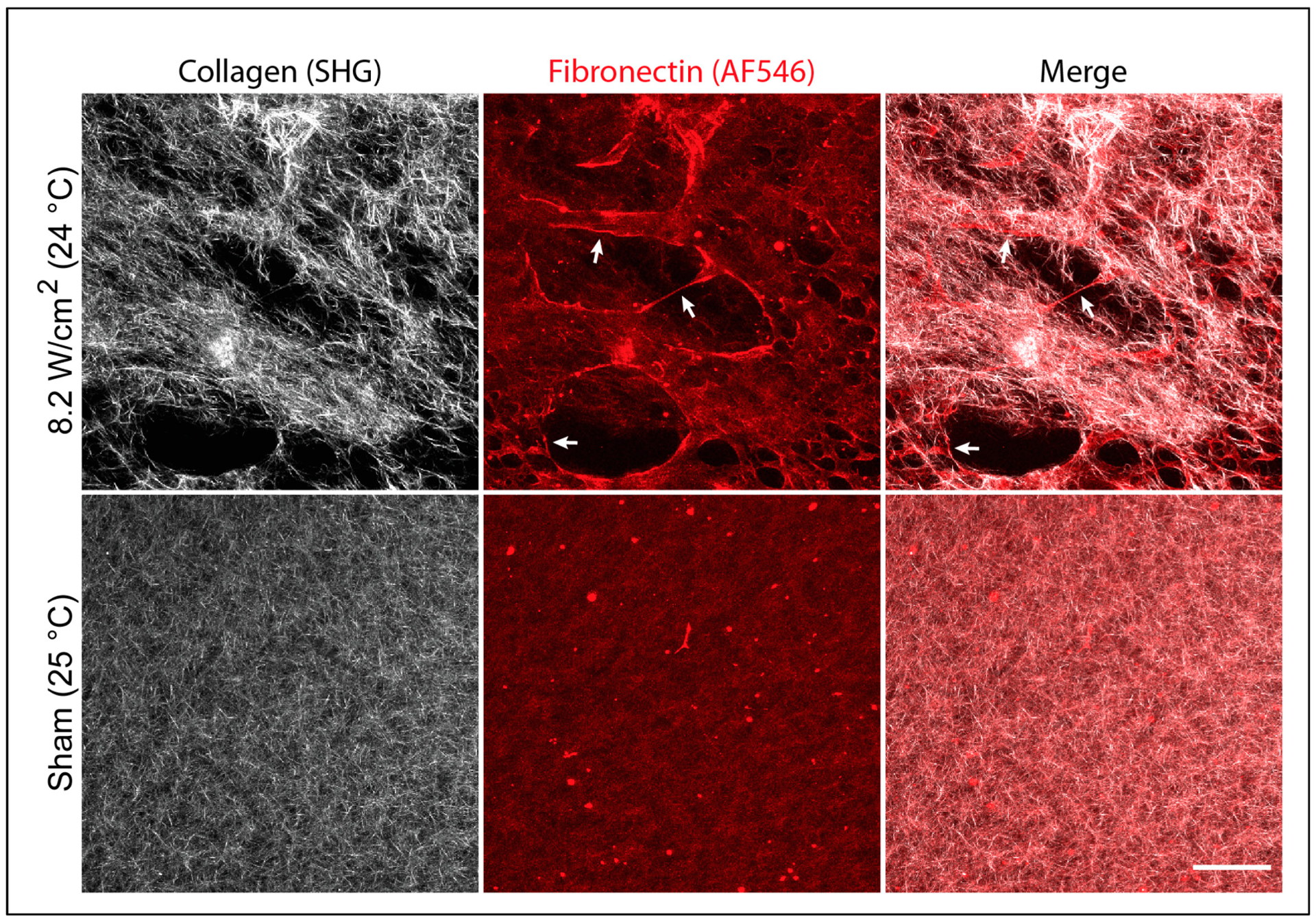
Mechanical effects of ultrasound trigger fibronectin fibril formation under permissive temperature conditions. Neutralized solutions of type I collagen (2 mg/mL) and fibronectin (25 μg/mL, 10% AF546-labeled) were co-exposed to either 8.8-MHz ultrasound at an intensity of 8.2 W/cm^2^ in an 18 °C water bath or sham-exposed in a 25 °C water bath such that the peak temperature achieved within each sample during exposure was 24–25 °C. Unbound fibronectin was removed by media exchange prior to fixation and multiphoton imaging of collagen (white) and fibronectin (red) and fiber structures. Maximum intensity z-projection images through a depth of 70 μm (5-μm z-steps) at the gel surface were assembled using FIJI software. Representative images from 1 of 3 samples per condition fabricated on independent days. Scale bar = 100 μm. Arrows indicate the presence of fibronectin fibrils.

To investigate non-thermal effects of ultrasound on fibronectin structure within 3D hydrogels, low-temperature, ultrasound-exposed (8.8 MHz, 8.2 W/cm^2^, 24 °C peak temperature) composite hydrogels were fabricated, and the structures of both fibronectin and collagen were visualized with multiphoton microscopy. Under these fabrication conditions, high-intensity fibrillar fibronectin staining was observed (Figure 6, 8.2 W/cm^2^, arrows). These fibronectin fibrils localized primarily to the periphery of pores outlined by dense collagen fibers, and bridged gaps between adjacent collagen fiber bundles (Figure 6, 8.2 W/cm^2^, Merge, arrows). In contrast, gels polymerized under temperature-matched, sham-exposure conditions exhibited diffuse, homogeneous fibronectin staining that largely co-localized with the underlying short collagen fibers, with no evidence of fibronectin fibril formation (Figure 6, Sham, Merge). Taken together, these results suggest that under permissive temperature conditions (e.g., <30 °C), ultrasound can influence the fibrillar structure of fibronectin within 3D collagen hydrogels via non-thermal mechanisms, possibly by providing sufficient mechanical force to initiate fibronectin fibril assembly.

## 4. Discussion

Previous work demonstrated that ultrasound can influence the microstructure of collagen via distinct thermal [27] and non-thermal mechanisms [28]. Here, acoustically modified hydrogels were manufactured under two sets of temperature conditions to assess the effects of ultrasound on collagen and fibronectin structure and function. Results show that exposing solutions of collagen and fibronectin to ultrasound during co-polymerization at temperatures greater than 30 °C produced composite hydrogels that supported accelerated microtissue formation and reduced cell adhesion. Similar effects were observed with composite hydrogels polymerized under temperature-matched sham conditions, indicating that the structural changes responsible for both accelerated microtissue formation and reduced adhesion are mediated by a thermal acoustic mechanism.

Acoustically modified composite hydrogels fabricated in the presence of ultrasound under temperature conditions below 30 °C also supported microtissue formation, but this was not observed on corresponding temperature-matched sham hydrogels (Figure 5). Multiphoton imaging of composite hydrogels polymerized under these reduced-temperature conditions revealed the formation of fibronectin fibrils at the hydrogel surface. The formation of fibronectin fibers was observed only on ultrasound-exposed gels polymerized under low-temperature conditions. In contrast, low-intensity fibronectin staining that co-localized diffusely with collagen fibers was observed under ultrasound exposure conditions in which temperature exceeded 30 °C (Figure 1) as well as temperature-matched sham gels (Figure 6). These data suggest that under permissive temperature conditions, ultrasound can influence fibronectin structure and function via a non-thermal acoustic mechanism.

Fibronectin fibril assembly by cells is a highly regulated process requiring both integrin ligation and cell-derived mechanical forces [13]. Several acellular techniques to produce artificial fibronectin fibrils have been reported, typically via the application of mechanical force at a fluid-air interface [24,38–40]. To the best of our knowledge, the importance of a fluid-air interface in acellular models of fibronectin fibril assembly has not been investigated systematically. However, application of mechanical forces that lead to exposure of cryptic fibronectin self-association sites is an essential step in the formation of both cell-derived and artificial fibronectin matrices [41]. Of note, the fibronectin fibers observed in the present study were primarily at or near the hydrogel surface boundary. As such, we speculate that ultrasound-driven fluid streaming at the fluid-air interface may be a mechanism to induce fibronectin self-association. Potential contributions of other non-thermal effects of ultrasound, such as acoustic cavitation, may also play a role in fibril formation by producing localized mechanical forces on collagen, fibronectin, or both proteins to trigger fibril formation.

Numerous additional mechanical and biochemical factors have the potential to influence fibronectin fibril assembly, including fibronectin conformation and collagen–fibronectin interactions [17,22,24]. In the present study, both thermal and non-thermal effects of ultrasound were observed on the fiber structure produced in collagen–fibronectin hydrogels. Critically, non-thermal effects of ultrasound were observed only under permissive temperature conditions. As solution temperatures are decreased below 30 °C, fibronectin undergoes a subtle conformational change that increases its affinity for collagen [22]. Thus, mechanical effects of ultrasound (e.g., fluid streaming) combined with the temperature-induced increase in binding affinity of collagen monomers to fibronectin’s collagen-binding domains [28], may serve to expose cryptic self-association sites in fibronectin to precipitate fibronectin fibril formation. The temperature-dependence of this effect has significant impact for future development of acoustically modified biomaterials for regenerative medicine applications, as it suggests the temperature at which materials are manufactured, either ex vivo or in situ, may offer an additional parameter by which to tune the structure and downstream cellular responses of manufactured materials in a therapeutic context. In summary, the combined results of these studies demonstrate the versatility of ultrasound as a technique for influencing the structure and function of collagen–fibronectin composite materials via distinct acoustic mechanisms.

## 5. Conclusions

The results of this study indicate that ultrasound exposure during fabrication of collagen–fibronectin composite gels can induce changes in the fiber structure of both components. Ultrasound-induced structural changes accelerated microtissue assembly. Furthermore, the effects of ultrasound were mediated through both thermal and non-thermal mechanisms, with evidence that the mechanical mechanisms associated with ultrasound propagation through a polymerizing ECM hydrogel structure can induce fibronectin fibril formation under permissive temperature conditions. Given the utility of both collagen and fibronectin as structure- and function-defining components of therapeutic wound dressings, this technology therefore holds significant potential as a non-invasive, site-specific technique to optimize materials for regenerative medicine applications.

## Figures and Tables

**Table 1.** Summary of acoustic exposure conditions. Ultrasound fields were generated using an unfocused piezoceramic transducer with diameter of 1 cm and a fundamental frequency of 8.8 MHz. Fields were calibrated using a capsule hydrophone at a position in the free field 10.5 cm from the surface of the transducer. The temperature within a polymerizing collagen sample (2 mg/mL) was measured under each set of exposure conditions using a wire thermocouple embedded at the center of the gel. Data are presented as mean ± SEM for *n* ≥ 9 (intensity and pressure) or *n* ≥ 3 (temperature) measurements per condition on independent experimental days.

Water Tank Temperature	Acoustic Intensity (I_SPPA_)	Peak Positive Pressure	Peak Negative Pressure	Final Temperature
18 °C	8.2 ± 0.1 W/cm^2^	0.76 ± 0.02 MPa	0.44 ± 0.02 MPa	24.2 ± 0.3 °C
25 °C	Sham (0 W/cm^2^)	-	-	24.6 ± 0.1 °C
25 °C	3.8 ± 0.1 W/cm^2^	0.48 ± 0.02 MPa	0.28 ± 0.02 MPa	27.7 ± 0.1 °C
25 °C	8.2 ± 0.1 W/cm^2^	0.76 ± 0.02 MPa	0.44 ± 0.02 MPa	32.3 ± 0.2 °C
37 °C	Sham (0 W/cm^2^)	-	-	34.5 ± 0.1 °C

## Featured Application:

Ultrasound is emerging as a versatile tool for manipulating scaffold structure in tissue engineering applications, as it can be applied non-invasively and site-specifically. Ultrasound fields are highly controllable, allowing for design of optimized exposure scenarios for a variety of systems and biomaterials. We envision ultrasound fields as a simple, direct, controllable, non-invasive, and non-toxic energy source to enable molecular control over the structural characteristics of extracellular matrix molecules to improve their biological function.
